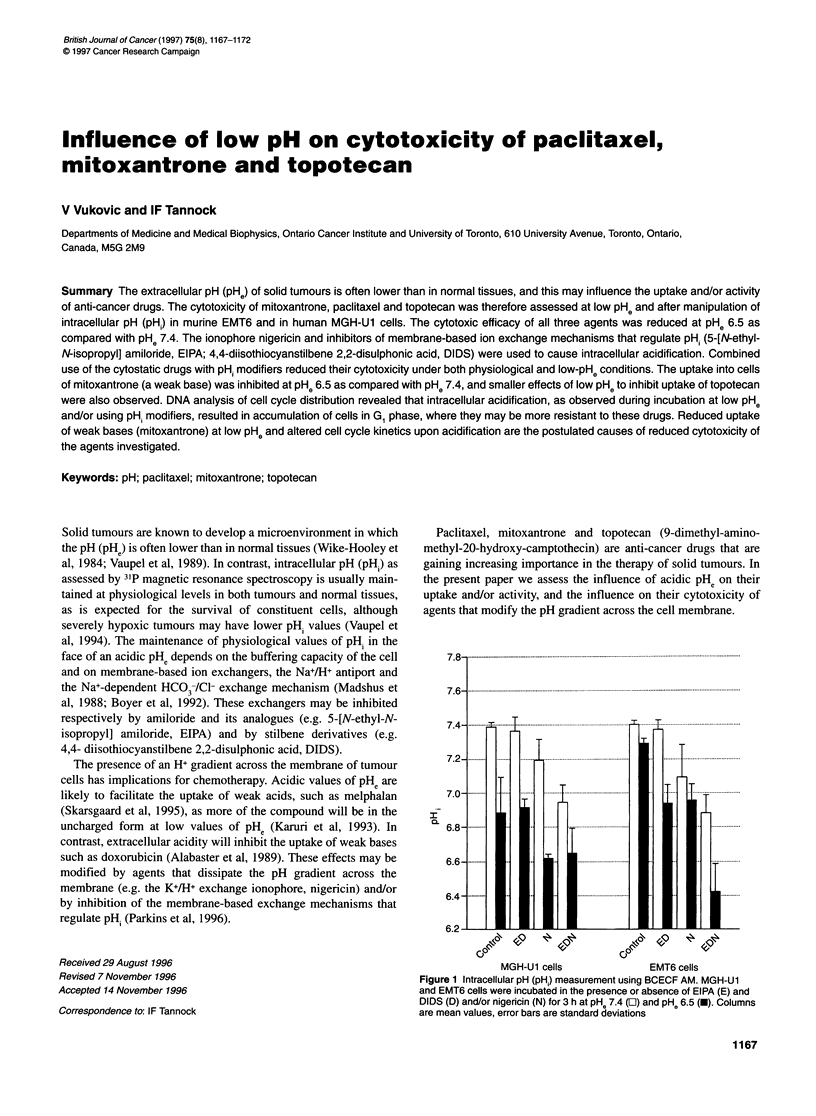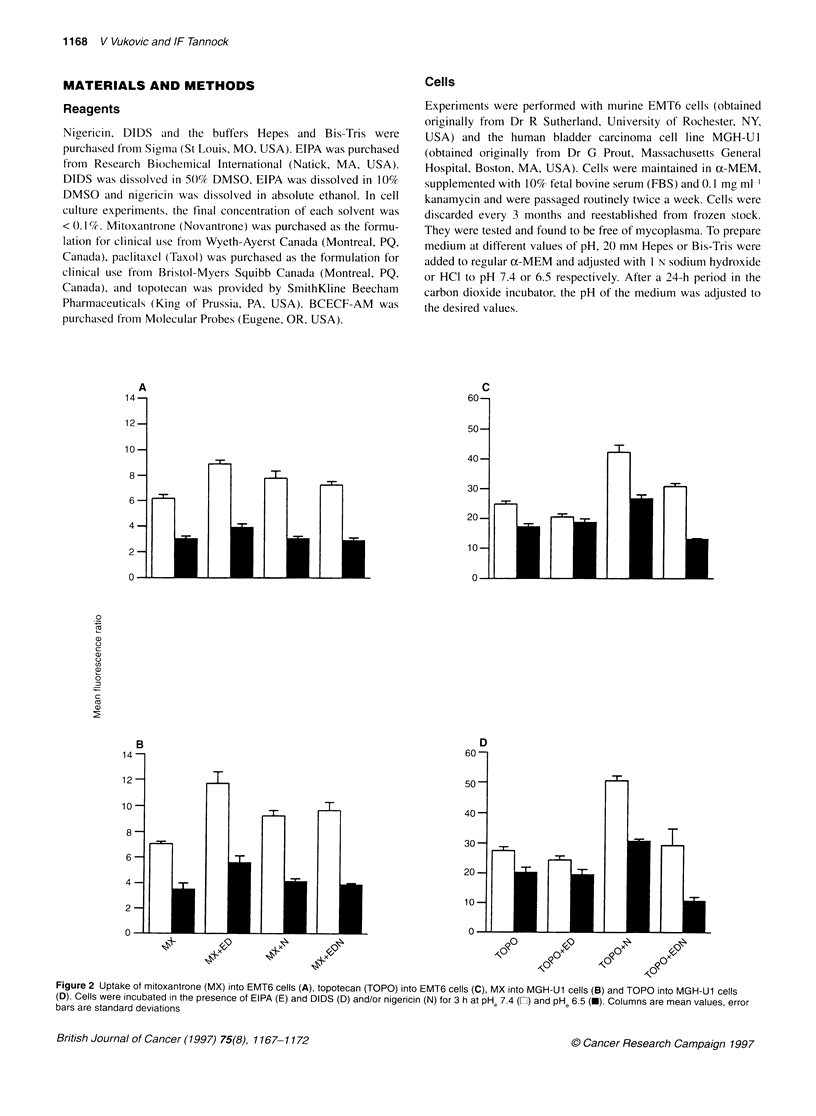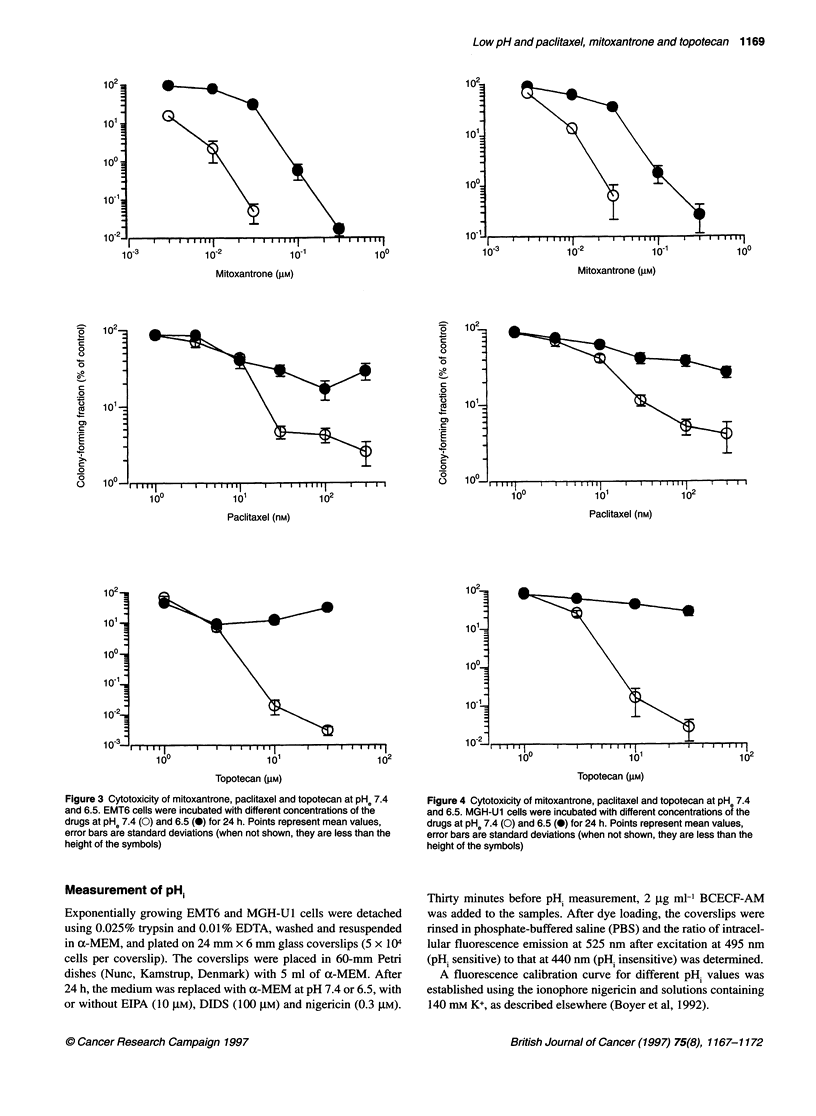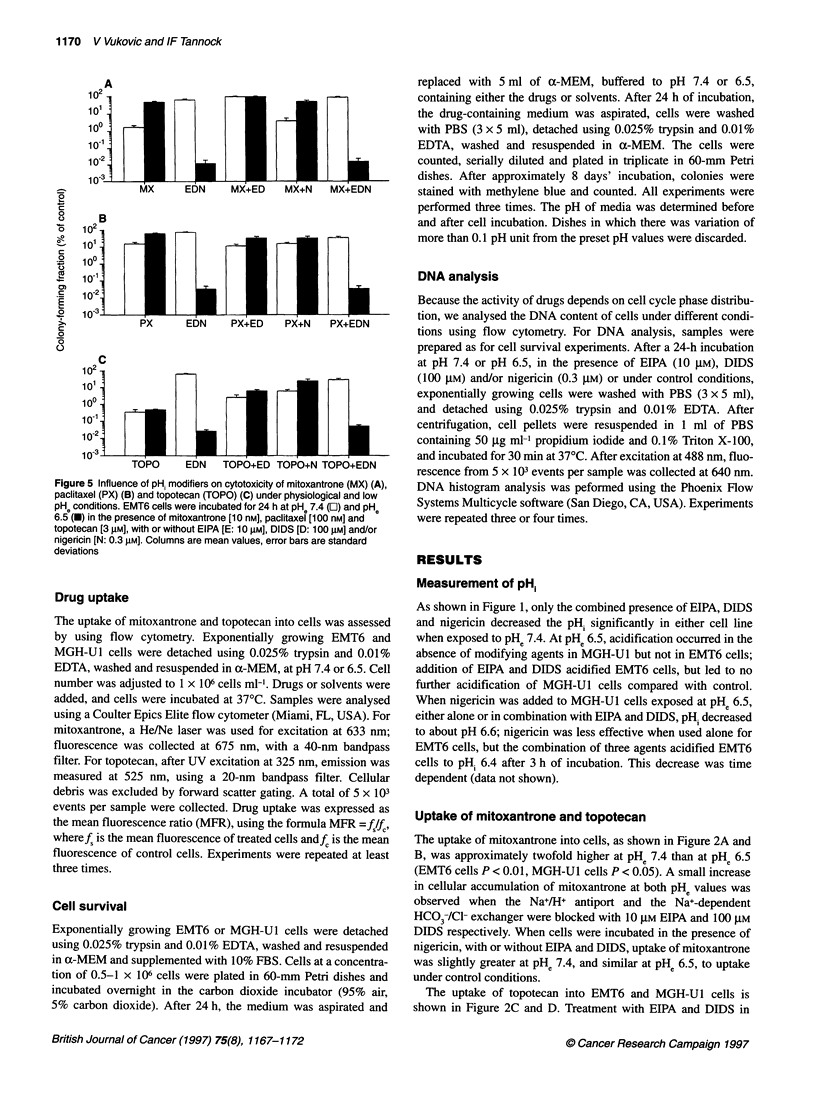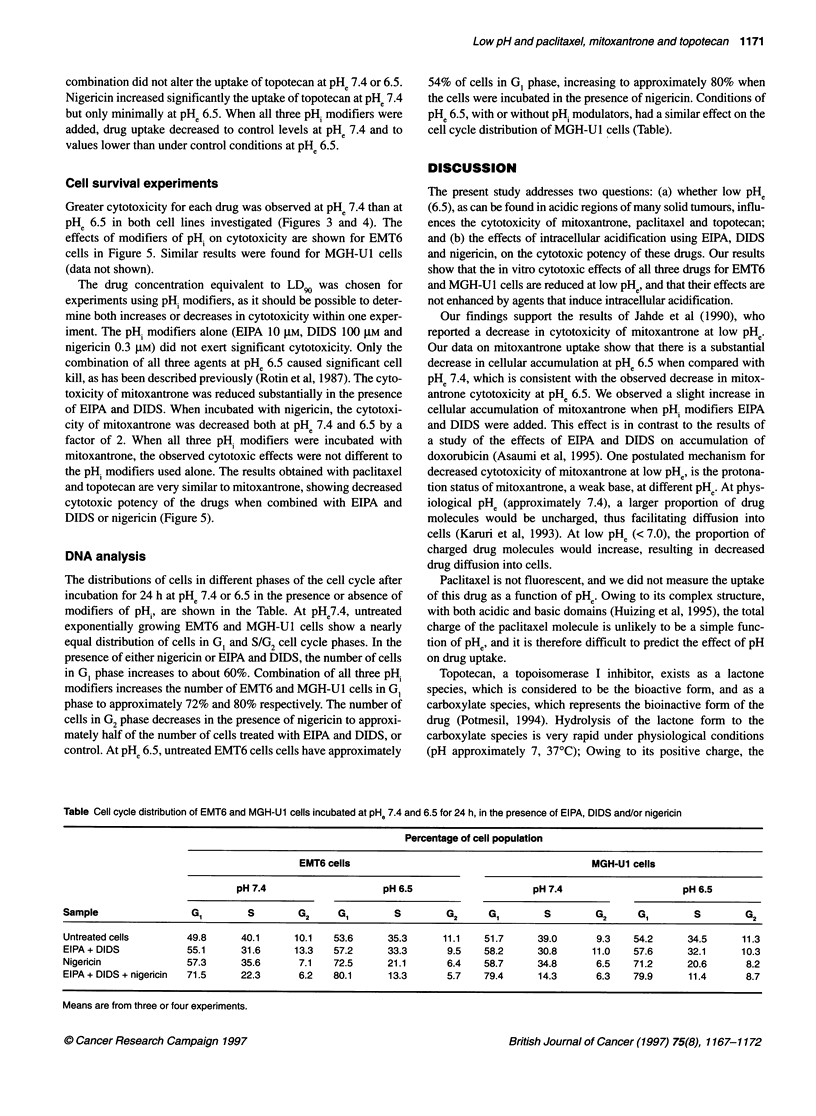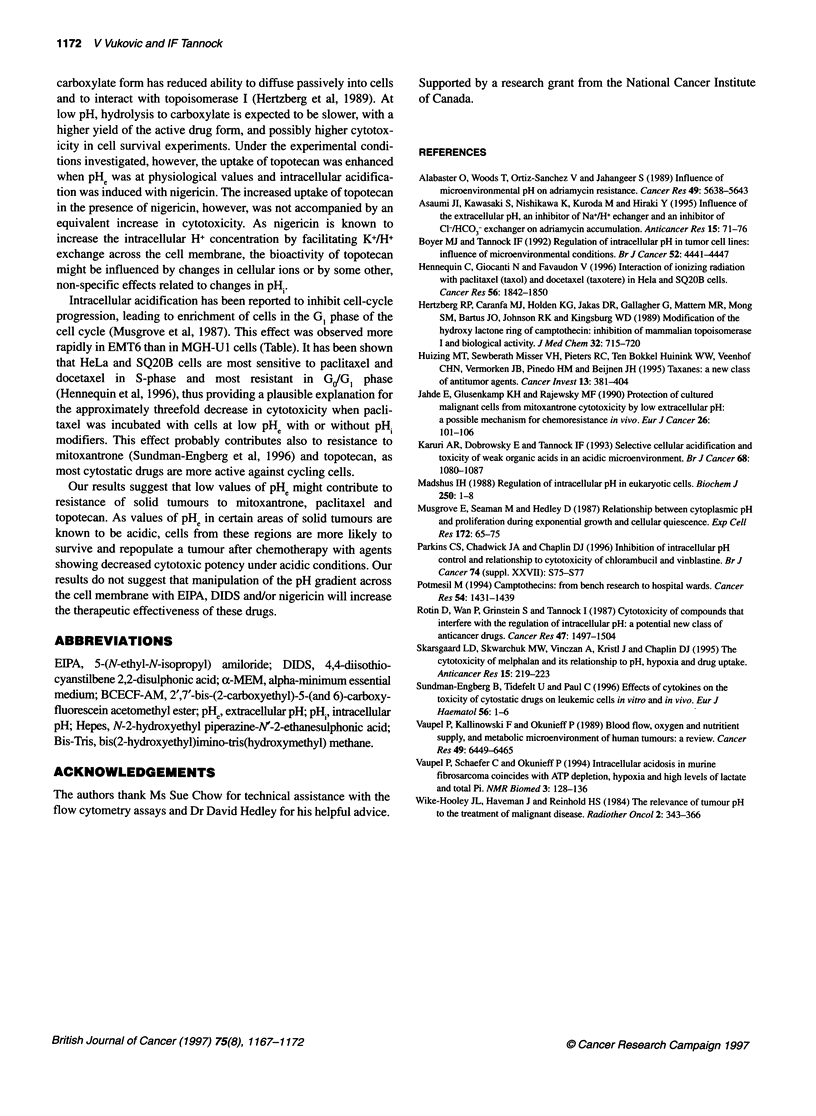# Influence of low pH on cytotoxicity of paclitaxel, mitoxantrone and topotecan.

**DOI:** 10.1038/bjc.1997.201

**Published:** 1997

**Authors:** V. Vukovic, I. F. Tannock

**Affiliations:** Department of Medicine, Ontario Cancer Institute and University of Toronto, Canada.

## Abstract

The extracellular pH (pHe) of solid tumours is often lower than in normal tissues, and this may influence the uptake and/or activity of anti-cancer drugs. The cytotoxicity of mitoxantrone, paclitaxel and topotecan was therefore assessed at low pHe and after manipulation of intracellular pH (pHi) in murine EMT6 and in human MGH-U1 cells. The cytotoxic efficacy of all three agents was reduced at pHe 6.5 as compared with pHe 7.4. The ionophore nigericin and inhibitors of membrane-based ion exchange mechanisms that regulate pHi (5-[N-ethyl-N-isopropyl] amiloride, EIPA; 4,4-diisothiocyanstilbene 2,2-disulphonic acid, DIDS) were used to cause intracellular acidification. Combined use of the cytostatic drugs with pHi modifiers reduced their cytotoxicity under both physiological and low-pHe conditions. The uptake into cells of mitoxantrone (a weak base) was inhibited at pHe 6.5 as compared with pHe 7.4, and smaller effects of low pHe to inhibit uptake of topotecan were also observed. DNA analysis of cell cycle distribution revealed that intracellular acidification, as observed during incubation at low pHe and/or using pHi modifiers, resulted in accumulation of cells in G1 phase, where they may be more resistant to these drugs. Reduced uptake of weak bases (mitoxantrone) at low pHe and altered cell cycle kinetics upon acidification are the postulated causes of reduced cytotoxicity of the agents investigated.


British Journal of Cancer (1997) 75(8), 1167–1172
© 1997 Cancer Research Campaign

### Influence of low pH on cytotoxicity of paclitaxel, mitoxantrone and topotecan

**V Vukovic and IF Tannock**

Departments of Medicine and Medical Biophysics, Ontario Cancer Institute and University of Toronto, 610 University Avenue, Toronto, Ontario, Canada, M5G 2M9

**Summary** The extracellular pH ($pH_e$) of solid tumours is often lower than in normal tissues, and this may influence the uptake and/or activity of anti-cancer drugs. The cytotoxicity of mitoxantrone, paclitaxel and topotecan was therefore assessed at low $pH_e$ and after manipulation of intracellular pH ($pH_i$) in murine EMT6 and in human MGH-U1 cells. The cytotoxic efficacy of all three agents was reduced at $pH_e$ 6.5 as compared with $pH_e$ 7.4. The ionophore nigericin and inhibitors of membrane-based ion exchange mechanisms that regulate $pH_i$ (5-[*N*-ethyl-*N*-isopropyl] amiloride, EIPA; 4,4-diisothiocyanstilbene 2,2-disulphonic acid, DIDS) were used to cause intracellular acidification. Combined use of the cytostatic drugs with $pH_i$ modifiers reduced their cytotoxicity under both physiological and low-$pH_e$ conditions. The uptake into cells of mitoxantrone (a weak base) was inhibited at $pH_e$ 6.5 as compared with $pH_e$ 7.4, and smaller effects of low $pH_e$ to inhibit uptake of topotecan were also observed. DNA analysis of cell cycle distribution revealed that intracellular acidification, as observed during incubation at low $pH_e$ and/or using $pH_i$ modifiers, resulted in accumulation of cells in $G_1$ phase, where they may be more resistant to these drugs. Reduced uptake of weak bases (mitoxantrone) at low $pH_e$ and altered cell cycle kinetics upon acidification are the postulated causes of reduced cytotoxicity of the agents investigated.

**Keywords:** pH; paclitaxel; mitoxantrone; topotecan

Solid tumours are known to develop a microenvironment in which the pH ($pH_e$) is often lower than in normal tissues (Wike-Hooley et al, 1984; Vaupel et al, 1989). In contrast, intracellular pH ($pH_i$) as assessed by $^{31}P$ magnetic resonance spectroscopy is usually maintained at physiological levels in both tumours and normal tissues, as is expected for the survival of constituent cells, although severely hypoxic tumours may have lower $pH_i$ values (Vaupel et al, 1994). The maintenance of physiological values of $pH_i$ in the face of an acidic $pH_e$ depends on the buffering capacity of the cell and on membrane-based ion exchangers, the $Na^+/H^+$ antiport and the $Na^+$-dependent $HCO_3^-/Cl^-$ exchange mechanism (Madshus et al, 1988; Boyer et al, 1992). These exchangers may be inhibited respectively by amiloride and its analogues (e.g. 5-[*N*-ethyl-*N*-isopropyl] amiloride, EIPA) and by stilbene derivatives (e.g. 4,4- diisothiocyanstilbene 2,2-disulphonic acid, DIDS).

The presence of an $H^+$ gradient across the membrane of tumour cells has implications for chemotherapy. Acidic values of $pH_e$ are likely to facilitate the uptake of weak acids, such as melphalan (Skarsgaard et al, 1995), as more of the compound will be in the uncharged form at low values of $pH_e$ (Karuri et al, 1993). In contrast, extracellular acidity will inhibit the uptake of weak bases such as doxorubicin (Alabaster et al, 1989). These effects may be modified by agents that dissipate the pH gradient across the membrane (e.g. the $K^+/H^+$ exchange ionophore, nigericin) and/or by inhibition of the membrane-based exchange mechanisms that regulate $pH_i$ (Parkins et al, 1996).

*Received 29 August 1996*
*Revised 7 November 1996*
*Accepted 14 November 1996*

*Correspondence to*: IF Tannock

Paclitaxel, mitoxantrone and topotecan (9-dimethyl-amino-methyl-20-hydroxy-camptothecin) are anti-cancer drugs that are gaining increasing importance in the therapy of solid tumours. In the present paper we assess the influence of acidic $pH_e$ on their uptake and/or activity, and the influence on their cytotoxicity of agents that modify the pH gradient across the cell membrane.

**Figure 1** Intracellular pH ($pH_i$) measurement using BCECF AM. MGH-U1 and EMT6 cells were incubated in the presence or absence of EIPA (E) and DIDS (D) and/or nigericin (N) for 3 h at $pH_e$ 7.4 (□) and $pH_e$ 6.5 (■). Columns are mean values, error bars are standard deviations

### MATERIALS AND METHODS

### Reagents

Nigericin, DIDS and the buffers Hepes and Bis-Tris were purchased from Sigma (St Louis, MO, USA). EIPA was purchased from Research Biochemical International (Natick, MA, USA). DIDS was dissolved in 50% DMSO, EIPA was dissolved in 10% DMSO and nigericin was dissolved in absolute ethanol. In cell culture experiments, the final concentration of each solvent was < 0.1%. Mitoxantrone (Novantrone) was purchased as the formulation for clinical use from Wyeth-Ayerst Canada (Montreal, PQ, Canada), paclitaxel (Taxol) was purchased as the formulation for clinical use from Bristol-Myers Squibb Canada (Montreal, PQ, Canada), and topotecan was provided by SmithKline Beecham Pharmaceuticals (King of Prussia, PA, USA). BCECF-AM was purchased from Molecular Probes (Eugene, OR, USA).

### Cells

Experiments were performed with murine EMT6 cells (obtained originally from Dr R Sutherland, University of Rochester, NY, USA) and the human bladder carcinoma cell line MGH-U1 (obtained originally from Dr G Prout, Massachusetts General Hospital, Boston, MA, USA). Cells were maintained in α-MEM, supplemented with 10% fetal bovine serum (FBS) and 0.1 mg $ml^{-1}$ kanamycin and were passaged routinely twice a week. Cells were discarded every 3 months and reestablished from frozen stock. They were tested and found to be free of mycoplasma. To prepare medium at different values of pH, 20 mM Hepes or Bis-Tris were added to regular α-MEM and adjusted with 1 N sodium hydroxide or HCl to pH 7.4 or 6.5 respectively. After a 24-h period in the carbon dioxide incubator, the pH of the medium was adjusted to the desired values.

**Figure 2** Uptake of mitoxantrone (MX) into EMT6 cells (**A**), topotecan (TOPO) into EMT6 cells (**C**), MX into MGH-U1 cells (**B**) and TOPO into MGH-U1 cells (**D**). Cells were incubated in the presence of EIPA (E) and DIDS (D) and/or nigericin (N) for 3 h at $pH_e$ 7.4 (□) and $pH_e$ 6.5 (■). Columns are mean values, error bars are standard deviations

© Cancer Research Campaign 1997

**Figure 3** Cytotoxicity of mitoxantrone, paclitaxel and topotecan at $pH_e$ 7.4 and 6.5. EMT6 cells were incubated with different concentrations of the drugs at $pH_e$ 7.4 (○) and 6.5 (●) for 24 h. Points represent mean values, error bars are standard deviations (when not shown, they are less than the height of the symbols)

**Figure 4** Cytotoxicity of mitoxantrone, paclitaxel and topotecan at $pH_e$ 7.4 and 6.5. MGH-U1 cells were incubated with different concentrations of the drugs at $pH_e$ 7.4 (○) and 6.5 (●) for 24 h. Points represent mean values, error bars are standard deviations (when not shown, they are less than the height of the symbols)

### Measurement of $pH_i$

Exponentially growing EMT6 and MGH-U1 cells were detached using 0.025% trypsin and 0.01% EDTA, washed and resuspended in α-MEM, and plated on 24 mm × 6 mm glass coverslips (5 × $10^4$ cells per coverslip). The coverslips were placed in 60-mm Petri dishes (Nunc, Kamstrup, Denmark) with 5 ml of α-MEM. After 24 h, the medium was replaced with α-MEM at pH 7.4 or 6.5, with or without EIPA (10 μM), DIDS (100 μM) and nigericin (0.3 μM). Thirty minutes before $pH_i$ measurement, 2 μg $ml^{-1}$ BCECF-AM was added to the samples. After dye loading, the coverslips were rinsed in phosphate-buffered saline (PBS) and the ratio of intracellular fluorescence emission at 525 nm after excitation at 495 nm ($pH_i$ sensitive) to that at 440 nm ($pH_i$ insensitive) was determined.

A fluorescence calibration curve for different $pH_i$ values was established using the ionophore nigericin and solutions containing 140 mM $K^+$, as described elsewhere (Boyer et al, 1992).

© Cancer Research Campaign 1997

**Figure 5** Influence of $pH_i$ modifiers on cytotoxicity of mitoxantrone (MX) (**A**), paclitaxel (PX) (**B**) and topotecan (TOPO) (**C**) under physiological and low $pH_e$ conditions. EMT6 cells were incubated for 24 h at $pH_e$ 7.4 (□) and $pH_e$ 6.5 (■) in the presence of mitoxantrone [10 nM], paclitaxel [100 nM] and topotecan [3 μM], with or without EIPA [E: 10 μM], DIDS [D: 100 μM] and/or nigericin [N: 0.3 μM]. Columns are mean values, error bars are standard deviations

### Drug uptake

The uptake of mitoxantrone and topotecan into cells was assessed by using flow cytometry. Exponentially growing EMT6 and MGH-U1 cells were detached using 0.025% trypsin and 0.01% EDTA, washed and resuspended in α-MEM, at pH 7.4 or 6.5. Cell number was adjusted to $1 \times 10^6$ cells $ml^{-1}$. Drugs or solvents were added, and cells were incubated at 37°C. Samples were analysed using a Coulter Epics Elite flow cytometer (Miami, FL, USA). For mitoxantrone, a He/Ne laser was used for excitation at 633 nm; fluorescence was collected at 675 nm, with a 40-nm bandpass filter. For topotecan, after UV excitation at 325 nm, emission was measured at 525 nm, using a 20-nm bandpass filter. Cellular debris was excluded by forward scatter gating. A total of $5 \times 10^3$ events per sample were collected. Drug uptake was expressed as the mean fluorescence ratio (MFR), using the formula MFR $= f_s/f_c$, where $f_s$ is the mean fluorescence of treated cells and $f_c$ is the mean fluorescence of control cells. Experiments were repeated at least three times.

### Cell survival

Exponentially growing EMT6 or MGH-U1 cells were detached using 0.025% trypsin and 0.01% EDTA, washed and resuspended in α-MEM and supplemented with 10% FBS. Cells at a concentration of 0.5–1 × $10^6$ cells were plated in 60-mm Petri dishes and incubated overnight in the carbon dioxide incubator (95% air, 5% carbon dioxide). After 24 h, the medium was aspirated and replaced with 5 ml of α-MEM, buffered to pH 7.4 or 6.5, containing either the drugs or solvents. After 24 h of incubation, the drug-containing medium was aspirated, cells were washed with PBS (3 × 5 ml), detached using 0.025% trypsin and 0.01% EDTA, washed and resuspended in α-MEM. The cells were counted, serially diluted and plated in triplicate in 60-mm Petri dishes. After approximately 8 days' incubation, colonies were stained with methylene blue and counted. All experiments were performed three times. The pH of media was determined before and after cell incubation. Dishes in which there was variation of more than 0.1 pH unit from the preset pH values were discarded.

### DNA analysis

Because the activity of drugs depends on cell cycle phase distribution, we analysed the DNA content of cells under different conditions using flow cytometry. For DNA analysis, samples were prepared as for cell survival experiments. After a 24-h incubation at pH 7.4 or pH 6.5, in the presence of EIPA (10 μM), DIDS (100 μM) and/or nigericin (0.3 μM) or under control conditions, exponentially growing cells were washed with PBS (3 × 5 ml), and detached using 0.025% trypsin and 0.01% EDTA. After centrifugation, cell pellets were resuspended in 1 ml of PBS containing 50 μg $ml^{-1}$ propidium iodide and 0.1% Triton X-100, and incubated for 30 min at 37°C. After excitation at 488 nm, fluorescence from $5 \times 10^3$ events per sample was collected at 640 nm. DNA histogram analysis was peformed using the Phoenix Flow Systems Multicycle software (San Diego, CA, USA). Experiments were repeated three or four times.

### RESULTS

### Measurement of $pH_i$

As shown in Figure 1, only the combined presence of EIPA, DIDS and nigericin decreased the $pH_i$ significantly in either cell line when exposed to $pH_e$ 7.4. At $pH_e$ 6.5, acidification occurred in the absence of modifying agents in MGH-U1 but not in EMT6 cells; addition of EIPA and DIDS acidified EMT6 cells, but led to no further acidification of MGH-U1 cells compared with control. When nigericin was added to MGH-U1 cells exposed at $pH_e$ 6.5, either alone or in combination with EIPA and DIDS, $pH_i$ decreased to about pH 6.6; nigericin was less effective when used alone for EMT6 cells, but the combination of three agents acidified EMT6 cells to $pH_i$ 6.4 after 3 h of incubation. This decrease was time dependent (data not shown).

### Uptake of mitoxantrone and topotecan

The uptake of mitoxantrone into cells, as shown in Figure 2A and B, was approximately twofold higher at $pH_e$ 7.4 than at $pH_e$ 6.5 (EMT6 cells $P < 0.01$, MGH-U1 cells $P < 0.05$). A small increase in cellular accumulation of mitoxantrone at both $pH_e$ values was observed when the $Na^+/H^+$ antiport and the $Na^+$-dependent $HCO_3^-/Cl^-$ exchanger were blocked with 10 μM EIPA and 100 μM DIDS respectively. When cells were incubated in the presence of nigericin, with or without EIPA and DIDS, uptake of mitoxantrone was slightly greater at $pH_e$ 7.4, and similar at $pH_e$ 6.5, to uptake under control conditions.

The uptake of topotecan into EMT6 and MGH-U1 cells is shown in Figure 2C and D. Treatment with EIPA and DIDS in

© Cancer Research Campaign 1997

combination did not alter the uptake of topotecan at $pH_e$ 7.4 or 6.5. Nigericin increased significantly the uptake of topotecan at $pH_e$ 7.4 but only minimally at $pH_e$ 6.5. When all three $pH_i$ modifiers were added, drug uptake decreased to control levels at $pH_e$ 7.4 and to values lower than under control conditions at $pH_e$ 6.5.

### Cell survival experiments

Greater cytotoxicity for each drug was observed at $pH_e$ 7.4 than at $pH_e$ 6.5 in both cell lines investigated (Figures 3 and 4). The effects of modifiers of $pH_i$ on cytotoxicity are shown for EMT6 cells in Figure 5. Similar results were found for MGH-U1 cells (data not shown).

The drug concentration equivalent to $LD_{90}$ was chosen for experiments using $pH_i$ modifiers, as it should be possible to determine both increases or decreases in cytotoxicity within one experiment. The $pH_i$ modifiers alone (EIPA 10 μM, DIDS 100 μM and nigericin 0.3 μM) did not exert significant cytotoxicity. Only the combination of all three agents at $pH_e$ 6.5 caused significant cell kill, as has been described previously (Rotin et al, 1987). The cytotoxicity of mitoxantrone was reduced substantially in the presence of EIPA and DIDS. When incubated with nigericin, the cytotoxicity of mitoxantrone was decreased both at $pH_e$ 7.4 and 6.5 by a factor of 2. When all three $pH_i$ modifiers were incubated with mitoxantrone, the observed cytotoxic effects were not different to the $pH_i$ modifiers used alone. The results obtained with paclitaxel and topotecan are very similar to mitoxantrone, showing decreased cytotoxic potency of the drugs when combined with EIPA and DIDS or nigericin (Figure 5).

### DNA analysis

The distributions of cells in different phases of the cell cycle after incubation for 24 h at $pH_e$ 7.4 or 6.5 in the presence or absence of modifiers of $pH_i$, are shown in the Table. At $pH_e$7.4, untreated exponentially growing EMT6 and MGH-U1 cells show a nearly equal distribution of cells in $G_1$ and $S/G_2$ cell cycle phases. In the presence of either nigericin or EIPA and DIDS, the number of cells in $G_1$ phase increases to about 60%. Combination of all three $pH_i$ modifiers increases the number of EMT6 and MGH-U1 cells in $G_1$ phase to approximately 72% and 80% respectively. The number of cells in $G_2$ phase decreases in the presence of nigericin to approximately half of the number of cells treated with EIPA and DIDS, or control. At $pH_e$ 6.5, untreated EMT6 cells cells have approximately 54% of cells in $G_1$ phase, increasing to approximately 80% when the cells were incubated in the presence of nigericin. Conditions of $pH_e$ 6.5, with or without $pH_i$ modulators, had a similar effect on the cell cycle distribution of MGH-U1 cells (Table).

### DISCUSSION

The present study addresses two questions: (a) whether low $pH_e$ (6.5), as can be found in acidic regions of many solid tumours, influences the cytotoxicity of mitoxantrone, paclitaxel and topotecan; and (b) the effects of intracellular acidification using EIPA, DIDS and nigericin, on the cytotoxic potency of these drugs. Our results show that the in vitro cytotoxic effects of all three drugs for EMT6 and MGH-U1 cells are reduced at low $pH_e$, and that their effects are not enhanced by agents that induce intracellular acidification.

Our findings support the results of Jahde et al (1990), who reported a decrease in cytotoxicity of mitoxantrone at low $pH_e$. Our data on mitoxantrone uptake show that there is a substantial decrease in cellular accumulation at $pH_e$ 6.5 when compared with $pH_e$ 7.4, which is consistent with the observed decrease in mitoxantrone cytotoxicity at $pH_e$ 6.5. We observed a slight increase in cellular accumulation of mitoxantrone when $pH_i$ modifiers EIPA and DIDS were added. This effect is in contrast to the results of a study of the effects of EIPA and DIDS on accumulation of doxorubicin (Asaumi et al, 1995). One postulated mechanism for decreased cytotoxicity of mitoxantrone at low $pH_e$, is the protonation status of mitoxantrone, a weak base, at different $pH_e$. At physiological $pH_e$ (approximately 7.4), a larger proportion of drug molecules would be uncharged, thus facilitating diffusion into cells (Karuri et al, 1993). At low $pH_e$ (< 7.0), the proportion of charged drug molecules would increase, resulting in decreased drug diffusion into cells.

Paclitaxel is not fluorescent, and we did not measure the uptake of this drug as a function of $pH_e$. Owing to its complex structure, with both acidic and basic domains (Huizing et al, 1995), the total charge of the paclitaxel molecule is unlikely to be a simple function of $pH_e$, and it is therefore difficult to predict the effect of pH on drug uptake.

Topotecan, a topoisomerase I inhibitor, exists as a lactone species, which is considered to be the bioactive form, and as a carboxylate species, which represents the bioinactive form of the drug (Potmesil, 1994). Hydrolysis of the lactone form to the carboxylate species is very rapid under physiological conditions (pH approximately 7, 37°C); Owing to its positive charge, the

**Table** Cell cycle distribution of EMT6 and MGH-U1 cells incubated at $pH_e$ 7.4 and 6.5 for 24 h, in the presence of EIPA, DIDS and/or nigericin

| | Percentage of cell population | | | | | | | | | | | |
|---|---|---|---|---|---|---|---|---|---|---|---|---|
| | EMT6 cells | | | | | | MGH-U1 cells | | | | | |
| | pH 7.4 | | | pH 6.5 | | | pH 7.4 | | | pH 6.5 | | |
| **Sample** | $G_1$ | S | $G_2$ | $G_1$ | S | $G_2$ | $G_1$ | S | $G_2$ | $G_1$ | S | $G_2$ |
| Untreated cells | 49.8 | 40.1 | 10.1 | 53.6 | 35.3 | 11.1 | 51.7 | 39.0 | 9.3 | 54.2 | 34.5 | 11.3 |
| EIPA + DIDS | 55.1 | 31.6 | 13.3 | 57.2 | 33.3 | 9.5 | 58.2 | 30.8 | 11.0 | 57.6 | 32.1 | 10.3 |
| Nigericin | 57.3 | 35.6 | 7.1 | 72.5 | 21.1 | 6.4 | 58.7 | 34.8 | 6.5 | 71.2 | 20.6 | 8.2 |
| EIPA + DIDS + nigericin | 71.5 | 22.3 | 6.2 | 80.1 | 13.3 | 5.7 | 79.4 | 14.3 | 6.3 | 79.9 | 11.4 | 8.7 |

Means are from three or four experiments.

carboxylate form has reduced ability to diffuse passively into cells and to interact with topoisomerase I (Hertzberg et al, 1989). At low pH, hydrolysis to carboxylate is expected to be slower, with a higher yield of the active drug form, and possibly higher cytotoxicity in cell survival experiments. Under the experimental conditions investigated, however, the uptake of topotecan was enhanced when $pH_e$ was at physiological values and intracellular acidification was induced with nigericin. The increased uptake of topotecan in the presence of nigericin, however, was not accompanied by an equivalent increase in cytotoxicity. As nigericin is known to increase the intracellular $H^+$ concentration by facilitating $K^+/H^+$ exchange across the cell membrane, the bioactivity of topotecan might be influenced by changes in cellular ions or by some other, non-specific effects related to changes in $pH_i$.

Intracellular acidification has been reported to inhibit cell-cycle progression, leading to enrichment of cells in the $G_1$ phase of the cell cycle (Musgrove et al, 1987). This effect was observed more rapidly in EMT6 than in MGH-U1 cells (Table). It has been shown that HeLa and SQ20B cells are most sensitive to paclitaxel and docetaxel in S-phase and most resistant in $G_0/G_1$ phase (Hennequin et al, 1996), thus providing a plausible explanation for the approximately threefold decrease in cytotoxicity when paclitaxel was incubated with cells at low $pH_e$ with or without $pH_i$ modifiers. This effect probably contributes also to resistance to mitoxantrone (Sundman-Engberg et al, 1996) and topotecan, as most cytostatic drugs are more active against cycling cells.

Our results suggest that low values of $pH_e$ might contribute to resistance of solid tumours to mitoxantrone, paclitaxel and topotecan. As values of $pH_e$ in certain areas of solid tumours are known to be acidic, cells from these regions are more likely to survive and repopulate a tumour after chemotherapy with agents showing decreased cytotoxic potency under acidic conditions. Our results do not suggest that manipulation of the pH gradient across the cell membrane with EIPA, DIDS and/or nigericin will increase the therapeutic effectiveness of these drugs.

### ABBREVIATIONS

EIPA, 5-(*N*-ethyl-*N*-isopropyl) amiloride; DIDS, 4,4-diisothiocyanstilbene 2,2-disulphonic acid; α-MEM, alpha-minimum essential medium; BCECF-AM, 2′,7′-bis-(2-carboxyethyl)-5-(and 6)-carboxyfluorescein acetomethyl ester; $pH_e$, extracellular pH; $pH_i$, intracellular pH; Hepes, *N*-2-hydroxyethyl piperazine-*N*′-2-ethanesulphonic acid; Bis-Tris, bis(2-hydroxyethyl)imino-tris(hydroxymethyl) methane.

### ACKNOWLEDGEMENTS

The authors thank Ms Sue Chow for technical assistance with the flow cytometry assays and Dr David Hedley for his helpful advice.

Supported by a research grant from the National Cancer Institute of Canada.

### REFERENCES

Alabaster O, Woods T, Ortiz-Sanchez V and Jahangeer S (1989) Influence of microenvironmental pH on adriamycin resistance. *Cancer Res* **49**: 5638–5643

Asaumi JI, Kawasaki S, Nishikawa K, Kuroda M and Hiraki Y (1995) Influence of the extracellular pH, an inhibitor of $Na^+/H^+$ echanger and an inhibitor of $Cl^-/HCO_3^-$ exchanger on adriamycin accumulation. *Anticancer Res* **15**: 71–76

Boyer MJ and Tannock IF (1992) Regulation of intracellular pH in tumor cell lines: influence of microenvironmental conditions. *Br J Cancer* **52**: 4441–4447

Hennequin C, Giocanti N and Favaudon V (1996) Interaction of ionizing radiation with paclitaxel (taxol) and docetaxel (taxotere) in Hela and SQ20B cells. *Cancer Res* **56**: 1842–1850

Hertzberg RP, Caranfa MJ, Holden KG, Jakas DR, Gallagher G, Mattern MR, Mong SM, Bartus JO, Johnson RK and Kingsburg WD (1989) Modification of the hydroxy lactone ring of camptothecin: inhibition of mammalian topoisomerase I and biological activity. *J Med Chem* **32**: 715–720

Huizing MT, Sewberath Misser VH, Pieters RC, Ten Bokkel Huinink WW, Veenhof CHN, Vermorken JB, Pinedo HM and Beijnen JH (1995) Taxanes: a new class of antitumor agents. *Cancer Invest* **13**: 381–404

Jahde E, Glusenkamp KH and Rajewsky MF (1990) Protection of cultured malignant cells from mitoxantrone cytotoxicity by low extracellular pH: a possible mechanism for chemoresistance *in vivo*. *Eur J Cancer* **26**: 101–106

Karuri AR, Dobrowsky E and Tannock IF (1993) Selective cellular acidification and toxicity of weak organic acids in an acidic microenvironment. *Br J Cancer* **68**: 1080–1087

Madshus IH (1988) Regulation of intracellular pH in eukaryotic cells. *Biochem J* **250**: 1–8

Musgrove E, Seaman M and Hedley D (1987) Relationship between cytoplasmic pH and proliferation during exponential growth and cellular quiescence. *Exp Cell Res* **172**: 65–75

Parkins CS, Chadwick JA and Chaplin DJ (1996) Inhibition of intracellular pH control and relationship to cytotoxicity of chlorambucil and vinblastine. *Br J Cancer* **74** (suppl. XXVII): S75–S77

Potmesil M (1994) Camptothecins: from bench research to hospital wards. *Cancer Res* **54**: 1431–1439

Rotin D, Wan P, Grinstein S and Tannock I (1987) Cytotoxicity of compounds that interfere with the regulation of intracellular pH: a potential new class of anticancer drugs. *Cancer Res* **47**: 1497–1504

Skarsgaard LD, Skwarchuk MW, Vinczan A, Kristl J and Chaplin DJ (1995) The cytotoxicity of melphalan and its relationship to pH, hypoxia and drug uptake. *Anticancer Res* **15**: 219–223

Sundman-Engberg B, Tidefelt U and Paul C (1996) Effects of cytokines on the toxicity of cytostatic drugs on leukemic cells *in vitro* and *in vivo*. *Eur J Haematol* **56**: 1–6

Vaupel P, Kallinowski F and Okunieff P (1989) Blood flow, oxygen and nutritient supply, and metabolic microenvironment of human tumours: a review. *Cancer Res* **49**: 6449–6465

Vaupel P, Schaefer C and Okunieff P (1994) Intracellular acidosis in murine fibrosarcoma coincides with ATP depletion, hypoxia and high levels of lactate and total Pi. *NMR Biomed* **3**: 128–136

Wike-Hooley JL, Haveman J and Reinhold HS (1984) The relevance of tumour pH to the treatment of malignant disease. *Radiother Oncol* **2**: 343–366

© Cancer Research Campaign 1997